# New noninvasive index for predicting liver fibrosis in Asian patients with chronic viral hepatitis

**DOI:** 10.1038/s41598-017-03589-w

**Published:** 2017-06-12

**Authors:** Hung-Wei Wang, Cheng-Yuan Peng, Hsueh-Chou Lai, Wen-Pang Su, Chia-Hsin Lin, Po-Heng Chuang, Sheng-Hung Chen, Ching-Hsiang Chen, Wei-Fan Hsu, Guan-Tarn Huang

**Affiliations:** 1 Division of Hepatogastroenterology, Department of Internal Medicine, China Medical University Hospital, Taichung, Taiwan; 2 School of Medicine, China Medical University, Taichung, Taiwan; 3 School of Chinese Medicine, China Medical University, Taichung, Taiwan

## Abstract

We developed an optimal noninvasive index comprising routine laboratory parameters for predicting cirrhosis in chronic hepatitis B (CHB) and chronic hepatitis C (CHC) patients. This study included 992 CHB patients and 1,284 CHC patients who received liver biopsy. We developed the new index, named modified Fibrosis-4 (mFIB-4) according to four independent variables of the model: age, aspartate aminotransferase (AST), alanine aminotransferase (ALT), and platelet count. The formula of the mFIB-4 index is 10 × Age(years) × AST(U/L)/Platelet count(10^9^/L) × ALT(U/L). For predicting cirrhosis, the bootstrap areas under the receiver operating characteristic curve for platelet count, AST/ALT ratio (AAR), AAR/platelet ratio index (AARPRI), AST/platelet ratio index (APRI), FIB-4, Pohl score, age-platelet (AP) index, Lok index, fibrosis quotient (FibroQ), and mFIB-4 were 0.7680, 0.7400, 0.8070, 0.6090, 0.7690, 0.6990, 0.7850, 0.7960, 0.8110, and 0.8070 in CHB patients, and 0.8170, 0.7210, 0.8400, 0.7310, 0.8310, 0.6730, 0.8220, 0.8440, 0.8570, and 0.8480 in CHC patients, respectively. FibroQ and mFIB-4 exhibited the highest diagnostic performance levels for liver cirrhosis in CHB and CHC despite the inclusion of the international normalised ratio in the formulation of FibroQ. Thus, mFIB-4 is a simple, inexpensive, and readily available method for assessing the liver fibrosis stage of Asian patients with CHB or CHC.

## Introduction

Chronic hepatitis B (CHB) and chronic hepatitis C (CHC) are global healthcare issues and critical causes of liver cirrhosis and hepatocellular carcinoma^1–3^. Histological staging of liver fibrosis is essential for CHB and CHC patients, not only for the treatment decision but also for prognostication^4,5^. Currently, liver biopsy remains the gold standard for assessing liver fibrosis. However, liver biopsy is an invasive method with some potentially serious complications, such as intra-abdominal bleeding, severe abdominal pain, or mortality^6–8^. Therefore, as alternatives to liver biopsy, many noninvasive methods and scoring systems have been developed to assess the stages and dynamic changes of liver fibrosis. Currently, several advanced imaging technologies, including transient elastography, acoustic radiation force impulse elastography, and magnetic resonance imaging elastography, are utilised to measure hepatic fibrosis^9–11^. However, they are costly and not widely utilised by medical institutions.

Previous studies have reported many noninvasive indices for predicting liver fibrosis, including platelet count^12^, aspartate aminotransferase/alanine aminotransferase (AST/ALT) ratio (AAR)^13^, AST/platelet ratio index (APRI)^14^, AAR/platelet ratio index (AARPRI)^15^, Fibrosis-4 (FIB-4)^16^, Pohl score^17^, age-platelet (AP) index^18^, fibrosis quotient (FibroQ)^19^, and Lok index^20^. For hepatitis B-related liver fibrosis (F2–F4 versus F0–F1), APRI and FIB-4 exhibited moderate sensitivity and accuracy, with the areas under the receiver operating characteristic curve (AUROCs) of 0.81 and 0.81, respectively^21,22^. However, APRI and FIB-4 are not suitable for evaluating improvement in liver fibrosis after antiviral therapy^23^. For hepatitis C-related cirrhosis (F4 versus F0–F3), FIB-4 and the Lok index appeared to be useful for evaluating Asian patients, with AUROCs of 0.833 and 0.847, respectively^24^. Other indices, such as APRI, showed moderate diagnostic accuracy for evaluating cirrhosis, with an AUROC of 0.83^25^.

In this study, we developed an optimal noninvasive index comprising routine laboratory parameters for predicting cirrhosis in chronic viral hepatitis and compared the diagnostic performance levels for different liver fibrosis stages between the new index and previously published indices.

## Results

### Study population and liver histological characteristics

This study included 992 CHB patients and 1,284 CHC patients who received liver biopsy, and their laboratory data within 7 days before biopsy were extracted. The median age of these CHB and CHC patients was 45 and 54 years, respectively. According to the meta-analysis virus hepatitis histological scoring system (METAVIR), 8, 280, 321, 134, and 249 CHB patients exhibited the fibrosis stages of F0, F1, F2, F3, and F4, respectively, and 2, 368, 493, 213, and 208 CHC patients respectively exhibited these stages. The baseline characteristics and laboratory data of patients are shown in Table 1. Among patients with liver cirrhosis (F4), those with CHB were significantly younger and predominantly male and had significantly lower AST and ALT levels, higher creatinine levels, and higher platelet counts than those with CHC (*p* < 0.0001, Supplementary Table S1).

**Table 1:** Baseline characteristics of CHB and CHC patients.

Variables Median (IQR) or n (%)	CHB (n = 992)	CHC (n = 1,284)
Age: years	45 (19)	54 (16)
Sex		
Female	252 (25.4)	663 (51.6)
Male	740 (74.6)	621 (48.4)
Liver stage by biopsy		
F0: no fibrosis	8 (0.8)	2 (0.2)
F1: portal fibrosis without septa	280 (28.2)	368 (28.7)
F2: portal fibrosis with few septa	321 (32.4)	493 (38.4)
F3: numerous septa without cirrhosis	134 (13.5)	213 (16.6)
F4: cirrhosis	249 (25.1)	208 (16.2)
Laboratory parameters		
AST: IU/L	50 (52)	57 (60)
ALT: IU/L	66 (94)	77 (90)
Total bilirubin: mg/dL	1.02 (0.53)	0.96 (0.44)
PT: INR	1.1 (0.1)	1.1 (0.1)
Platelet: ×10^3^/μL	174 (78.5)	163 (81.5)
Creatinine: mg/dL	0.88 (0.3)	0.79 (0.27)

ALT, alanine aminotransferase; AST, aspartate aminotransferase; CHB, chronic hepatitis B; CHC, chronic hepatitis C; INR, international normalised ratio; PT, prothrombin time.

### New noninvasive index for liver cirrhosis

Based on the analysis of this cohort of CHB and CHC patients, five variables, namely age, AST, ALT, international normalised ratio (INR), and platelet count, were significantly associated with cirrhosis in the univariable logistic regression analysis (*p* < 0.001, Supplementary Table S2). INR exhibited a much higher weight of odds ratio because of its small absolute value (Supplementary Table S2). It was therefore not selected as a parameter in the multivariable model. Examination of the regression formula revealed that age, AST, ALT, and platelet count had a similar weight, with corresponding odds ratios of approximately 1.0 for both CHB and CHC patients (Table 2). Therefore, we constructed a new model for predicting cirrhosis and named this new index as modified FIB-4 (mFIB-4), because these two indices comprise the same parameters with a similar mathematical relationship.

$${\rm{m}}{\rm{F}}{\rm{I}}{\rm{B}}{\textstyle \text{-}}4=\frac{10\,\times \,{\rm{A}}{\rm{g}}{\rm{e}}\,({\rm{y}}{\rm{e}}{\rm{a}}{\rm{r}}{\rm{s}})\,\times \,{\rm{A}}{\rm{S}}{\rm{T}}\,({\rm{U}}/{\rm{L}})}{{\rm{P}}{\rm{l}}{\rm{a}}{\rm{t}}{\rm{e}}{\rm{l}}{\rm{e}}{\rm{t}}\,{\rm{c}}{\rm{o}}{\rm{u}}{\rm{n}}{\rm{t}}\,({10}^{9}/{\rm{L}})\,\times \,{\rm{A}}{\rm{L}}{\rm{T}}\,({\rm{U}}/{\rm{L}})}$$

**Table 2:** Multivariable logistic regression model of factors associated with cirrhosis in CHB and CHC patients.

Variables	CHB patients (n = 992)		CHC patients (n = 1,284)	
	OR (95% CI)	*p* value	OR (95% CI)	*p* value
Age: years	1.040 (1.025–1.056)	<0.0001	1.059 (1.038–1.081)	<0.0001
AST: IU/L	1.009 (1.004–1.013)	0.0002	1.018 (1.010–1.026)	<0.0001
ALT: IU/L	0.989 (0.984–0.993)	<0.0001	0.983 (0.977–0.990)	<0.0001
Platelet: ×10^3^/μL	0.982 (0.979–0.986)	<0.0001	0.974 (0.969–0.979)	<0.0001

ALT, alanine aminotransferase; AST, aspartate aminotransferase; CHB, chronic hepatitis B; CHC, chronic hepatitis C.

The diagnostic performance levels of the multivariable logistic regression model comprising age, ASL, ALT, and platelet count and the derived mFIB-4 index for cirrhosis were 0.8486 (95% confidence interval (CI): 0.8208–0.8765) and 0.8508 (95% CI: 0.8244–0.8773) for CHB and 0.8765 (95% CI: 0.8510–0.9021) and 0.8813 (95% CI: 0.8577–0.9049) for CHC, respectively (Table 3). INR included in the multivariable logistic regression model or its derived FibroQ index exhibited similar diagnostic performance for cirrhosis compared with that of the mFIB-4 index (Table 3). Moreover, INR may not be a routine laboratory test in daily practice. For these three reasons, we propose to adopt mFIB-4 as an inexpensive routine index for predicting liver cirrhosis in CHB and CHC.

**Table 3:** Models with different combinations of variables for predicting cirrhosis in CHB and CHC patients.

Variables in the model	CHB patients (n = 992)	CHC patients (n = 1,284)
	AUROC (95% CI)	AUROC (95% CI)
Age, AST, ALT, Platelets	0.8486 (0.8208–0.8765)	0.8765 (0.8510–0.9021)
Age, AST, ALT, Platelets, INR	0.8581 (0.8313–0.8849)	0.8893 (0.8862–0.9124)
mFIB-4	0.8508 (0.8244–0.8773)	0.8813 (0.8577–0.9049)
FibroQ	0.8552 (0.8294–0.8809)	0.8901 (0.8679–0.9124)

ALT, alanine aminotransferase; AST, aspartate aminotransferase; CHB, chronic hepatitis B; CHC, chronic hepatitis C; FibroQ, fibrosis quotient; INR, international normalised ratio; mFIB-4, modified FIB-4.

### Values of noninvasive indices and their correlations with fibrosis stages

The median values of the various noninvasive indices for each liver fibrosis stage are shown in Table 4. Fibrosis indices, including AAR, AARPRI, APRI, FIB-4, AP index, Lok index, FibroQ, and mFIB-4, exhibited positive linear correlations (*p* < 0.0001) with the METAVIR fibrosis stages in CHB and CHC patients. Only the platelet count exhibited a negative linear correlation (*p* < 0.0001) with the METAVIR fibrosis stage (Table 4).

**Table 4:** Values of various noninvasive indices and their correlations with fibrosis stages.

Indices	METAVIR fibrosis stage				r_s_	*p* value
	1	2	3	4		
CHB patients n (%)	280 (28.2%)	321 (32.4%)	134 (13.5%)	249 (25.1%)		
Platelet count	196 (54.5)	189 (62.0)	150 (72)	117 (75)	−0.4904	<0.0001
AAR	0.62 (0.30)	0.67 (0.37)	0.86 (0.37)	1.05 (0.51)	0.4330	<0.0001
AARPRI	0.46 (0.32)	0.57 (0.39)	0.87 (0.62)	1.32 (1.50)	0.5610	<0.0001
APRI	0.63 (0.80)	0.78 (0.83)	0.93 (1.26)	1.03 (1.13)	0.2259	<0.0001
FIB-4	1.13 (0.83)	1.51 (1.22)	2.17 (1.69)	3.47 (3.55)	0.5169	<0.0001
AP index	3 (2.5)	4 (4)	6 (3)	7 (2)	0.5267	<0.0001
Lok index	0.28 (0.20)	0.34 (0.21)	0.53 (0.31)	0.68 (0.33)	0.5003	<0.0001
FibroQ	1.24 (1.13)	1.75 (1.86)	2.82 (2.95)	5.54 (7.29)	0.5333	<0.0001
mFIB-4	1.18 (1.12)	1.67 (1.68)	2.60 (2.61)	5.00 (5.91)	0.5297	<0.0001
**CHC patients n (%)**	**368 (28.7%)**	**493 (38.4%)**	**213 (16.6%)**	**208 (16.2%)**	**r**_**s**_	***p*** **value**
Platelet count	202 (74)	165 (60)	138 (61)	103 (50)	−0.5614	<0.0001
AAR	0.77 (0.39)	0.74 (0.29)	0.83 (0.34)	0.99 (0.43)	0.2365	<0.0001
AARPRI	0.57 (0.35)	0.68 (0.39)	0.92 (0.63)	1.56 (1.11)	0.5132	<0.0001
APRI	0.43 (0.50)	0.90 (0.94)	1.59 (1.67)	1.92 (2.07)	0.5190	<0.0001
FIB-4	1.29 (1.09)	2.11 (1.72)	3.33 (3.13)	5.36 (4.45)	0.6329	<0.0001
AP index	4 (3)	6 (3)	7 (3)	8 (2)	0.5759	<0.0001
Lok index	0.27 (0.16)	0.36 (0.20)	0.52 (0.28)	0.65 (0.25)	0.4742	<0.0001
FibroQ	1.79 (1.65)	2.48 (1.84)	3.86 (3.17)	6.80 (5.30)	0.4980	<0.0001
mFIB-4	1.78 (1.75)	2.36 (1.78)	3.52 (2.97)	6.18 (4.37)	0.4866	<0.0001

AAR, AST/ALT ratio; AARPRI, AAR/PLT ratio index; AP index, age-PLT index; APRI, AST/PLT ratio index; CHB, chronic hepatitis B; CHC, chronic hepatitis C; FIB-4, fibrosis index based on the four factors; FibroQ, fibrosis quotient; mFIB-4, modified FIB-4; r_s_: Spearman’s ranked correlation coefficients.

### Comparison of diagnostic performance levels of noninvasive indices for liver cirrhosis (F0–F3 versus F4)

ROC analysis revealed comparable diagnostic performance levels for the platelet count, AAR, AARPRI, APRI, FIB-4, Pohl score, AP index, Lok index, FibroQ, and mFIB-4 for the prediction of cirrhosis (F4), with bootstrap AUROCs of 0.7680, 0.7400, 0.8070, 0.6090, 0.7690, 0.6990, 0.7850, 0.7960, 0.8110, and 0.8070 in CHB patients and those of 0.8170, 0.7210, 0.8400, 0.7310, 0.8310, 0.6730, 0.8220, 0.8440, 0.8570, and 0.8480 in CHC patients, respectively (Table 5). FibroQ, AARPRI, and mFIB-4 exhibited the highest diagnostic performance levels for cirrhosis, with bootstrap AUROCs of >0.80, compared with those of other indices in CHB patients. The AUROC of mFIB-4 was not significantly different from those of FibroQ and AARPRI but was significantly higher than those of other indices (*p* < 0.05, Table 5). In CHC patients, FibroQ, mFIB-4, and Lok index exhibited the highest diagnostic performance levels, with bootstrap AUROCs of >0.84. The AUROC of mFIB-4 was not significantly different from those of FibroQ and Lok index but was significantly higher than those of other indices (*p* < 0.05, Table 5). Therefore, compared with other indices, FibroQ and mFIB-4 were the two optimal predictive indices for HBV- and HCV-related cirrhosis. The optimal cut-off values for the platelet count, AAR, AARPRI, APRI, FIB-4, AP index, Lok index, FibroQ, and mFIB-4 for predicting cirrhosis were 140, 0.8, 0.9, 0.7, 2.2, 6, 0.46, 3.3, and 2.9 for CHB and 130, 0.8, 1.0, 1.3, 3.8, 8, 0.52, 4.3, and 4.0 for CHC, respectively (Table 5). In CHB patients, the sensitivities of these indices were between 43% and 79.9%, and their specificities were between 48.4% and 93.8%. In CHC patients, the sensitivities of these indices were between 40.9% and 82.2%, and their specificities were between 56.2% and 92.2% (Table 5).

**Table 5:** Diagnostic accuracies of noninvasive indices for predicting cirrhosis (F0–F3 versus F4).

Index	AUROC	Cut-off	Sensitivity	Specificity	PPV	NPV	Sensitivity + Specificity-1	Bootstrap AUROC
CHB								
Platelet	0.8010 (0.8092–0.8346)	140	65.5%	82.9%	56.2%	87.8%	48.4%	0.7680
AAR	0.7763 (0.7111–0.8076)	0.8	77.5%	66.6%	43.8%	89.8%	44.1%	0.7400
AARPRI	0.8475 (0.8448–0.8742)^*^	0.9	72.3%	81.1%	56.3%	89.7%	53.4%	0.8070
APRI	0.6224 (0.7115–0.6605)	0.7	74.3%	48.4%	32.6%	84.9%	22.7%	0.6090
FIB-4	0.8053 (0.8344–0.8362)	2.2	72.7%	75.7%	50.1%	89.2%	48.4%	0.7690
Pohl score	0.6839 (0.6309–0.7159)	0,1	43.0%	93.8%	69.9%	83.1%	36.8%	0.6990
AP index	0.8152 (0.8153–0.8445)	6	75.5%	72.5%	48.0%	89.8%	48.0%	0.7850
Lok index	0.8332 (0.8466–0.8614)^*^	0.46	79.9%	73.3%	50.1%	91.6%	53.2%	0.7960
FibroQ	0.8552 (0.8578–0.8868)^*^	3.3	74.7%	79.3%	54.7%	90.3%	54.0%	0.8110
mFIB-4	0.8508 (0.8577–0.8773)^*^	2.9	75.5%	78.1%	53.7%	90.5%	53.6%	0.8070
CHC								
Platelet	0.8410 (0.8092–0.8729)	130	78.9%	78.4%	41.4%	95.1%	57.3%	0.8170
AAR	0.7454 (0.7111–0.7798)	0.8	82.2%	56.2%	26.6%	94.2%	38.4%	0.7210
AARPRI	0.8704 (0.8448–0.8959)	1	79.3%	78.9%	42.1%	95.2%	58.2%	0.8400
APRI	0.7473 (0.7115–0.7831)	1.3	70.2%	69.9%	31.1%	92.4%	40.1%	0.7310
FIB-4	0.8595 (0.8344–0.8847)	3.8	72.1%	83.7%	46.2%	94.0%	55.9%	0.8310
Pohl score	0.6653 (0.6309–0.6997)	0,1	40.9%	92.2%	50.3%	89.0%	33.1%	0.6730
AP index	0.8424 (0.8153–0.8695)	8	73.6%	80.8%	42.5%	94.1%	54.3%	0.8220
Lok index	0.8715 (0.8466–0.8965)^*^	0.52	78.9%	81.9%	45.7%	95.2%	60.7%	0.8440
FibroQ	0.8901 (0.8578–0.9224)^*^	4.3	78.9%	82.9%	47.1%	95.3%	61.8%	0.8570
mFIB-4	0.8813 (0.8577–0.9049)^*^	4.0	78.4%	81.5%	45.0%	95.1%	59.9%	0.8480

AAR, AST/ALT ratio; AARPRI, AAR/PLT ratio index; AP index, age-PLT index; APRI, AST/PLT ratio index; CHB, chronic hepatitis B; CHC, chronic hepatitis C; FIB-4, fibrosis index based on the four factors; FibroQ, fibrosis quotient; mFIB-4, modified FIB-4; NPV, negative predictive value; PPV, positive predictive value. *p > 0.05 compared with mFIB-4, *p* < 0.05 for all other indexes compared with mFIB-4.

### Comparison of diagnostic performance levels of noninvasive indices for advanced fibrosis (F0–F2 versus F3–F4)

The AUROCs were analysed to compare the diagnostic performance levels of the noninvasive indices for predicting advanced fibrosis (F3) and cirrhosis (F4). AUROCs for CHB patients using the platelet count, AAR, AARPRI, APRI, FIB-4, Pohl score, AP index, Lok index, FibroQ, and mFIB-4 were 0.7937, 0.7521, 0.8290, 0.6191, 0.7869, 0.6466, 0.8014, 0.8310, 0.8318, and 0.8232, respectively, whereas those for CHC patients were 0.7999, 0.6697, 0.8028, 0.7729, 0.8425, 0.6265, 0.8038, 0.8397, 0.8301, and 0.8152, respectively (Supplementary Table S3). For CHB, FibroQ, Lok index, and AARPRI exhibited the highest diagnostic performance levels compared with those of other indices. For CHC, FIB-4, Lok index, and FibroQ exhibited the highest diagnostic performance levels compared with those of other indices (Supplementary Table S3).

### Comparison of diagnostic performance levels of noninvasive indices for significant fibrosis (F0–F1 versus F2–F4)

We used AUROC to analyse the noninvasive indices for predicting significant fibrosis (F2–F4). AUROCs of the noninvasive indices, including platelet count, AAR, AARPRI, APRI, FIB-4, Pohl score, AP index, Lok index, FibroQ, and mFIB-4, were 0.6962, 0.6803, 0.7349, 0.6144, 0.7367, 0.5793, 0.7244, 0.7443, 0.7466, and 0.7390 in CHB patients and 0.7741, 0.5462, 0.7086, 0.7793, 0.8136, 0.5659, 0.7823, 0.7691, 0.7496, and 0.7368 in CHC patients, respectively (Supplementary Table S4). For CHB, FibroQ, Lok index, and mFIB-4 exhibited the highest diagnostic performance levels compared with those of other indices. For CHC, FIB-4, AP index, and APRI exhibited the highest diagnostic performance levels compared with those of other indices (Supplementary Table S4).

## Discussion

The identification of CHB and CHC patients with liver cirrhosis through liver biopsy is essential for clinical decisions such as whether to implement endoscopic screening for varices and determining the surveillance frequency for hepatocellular carcinoma. However, liver biopsy is invasive and potentially risky, particularly for patients with decompensated liver disease. Through comprehensive statistical analysis, we constructed a new noninvasive fibrosis index, mFIB-4, and demonstrated its high diagnostic performance for cirrhosis in CHB and CHC patients by comparing it with some previously reported noninvasive indices.

Previous studies have not demonstrated a well-established index for HBV-related fibrosis or cirrhosis. Shin *et al*. demonstrated that APRI exhibits higher performance (AUROC: 0.86) for predicting significant fibrosis (F2–F4) than other markers (API, AAR, and platelet count) in CHB^26^. In the Chronic Hepatitis Cohort study, Teshale *et al*. reported that APRI and FIB-4 exhibit high sensitivity and specificity for distinguishing F2–F4 from F0–F1, with AUROCs of 0.81 (0.76–0.87) and 0.81 (0.75–0.86), respectively^21^. Zhang *et al*. analysed three noninvasive models (FIB-4, APRI, and AAR) in 1,543 patients with HBV infection to predict cirrhosis and obtained adjusted AUROCs of 0.786, 0.710, and 0.644 for these models, respectively^27^. However, a recent meta-analysis involving 39 studies that detected HBV-related liver fibrosis revealed that the summary AUROC values of APRI and FIB-4 were 0.73 and 0.81 for advanced fibrosis and 0.73 and 0.84 for cirrhosis, respectively. It was concluded that APRI and FIB-4 exhibit only moderate sensitivity and accuracy for identifying liver fibrosis in CHB patients^22^. Our comparative study demonstrated that FibroQ, mFIB-4, and AARPRI exhibited the highest diagnostic performance levels (bootstrap AUROCs >0.80) for predicting HBV-related cirrhosis, and that both FibroQ and mFIB-4 exhibited significantly higher performance levels than those of FIB-4 and APRI (Table 5).

Several studies have indicated that APRI and FIB-4 exhibit high reliability for predicting liver fibrosis in CHC^24,25,28–30^. In a large US cohort of HCV-infected patients, FIB-4 exhibited significantly higher diagnostic accuracy than APRI for differentiating severe fibrosis (stages F3–F4) from mild-to-moderate fibrosis (stages F0–F2) (AUROC: 0.83 versus 0.80) and for predicting cirrhosis (AUROC: 0.8598 versus 0.8148)^29^. FIB-4 exhibited high diagnostic accuracy and utility for assessing different fibrosis stages in Asian patients with hepatitis C (AUROC: 0.833–0.871)^24^. Nonetheless, the Lok index had the highest AUROC (0.847) for predicting liver cirrhosis (F4). We observed that FibroQ, mFIB-4, Lok index, and AARPRI exhibited high diagnostic performance levels for predicting HCV-related liver cirrhosis (bootstrap AUROCs >0.84), and that both FibroQ and mFIB-4 exhibited significantly higher performance levels than those of FIB-4 and APRI (Table 5).

The AST/ALT ratio has been shown to be associated with the severity of fibrosis in patients with liver diseases of different aetiologies^13,31^. An AST/ALT ratio of ≥1.0 strongly suggests the presence of cirrhosis^32^. The platelet count has also been shown to be correlated with the degree of portal hypertension and advanced fibrosis^14,33^. Indices incorporating both AST/ALT and platelet count, such as FibroQ, mFIB-4, Lok index, AARPRI, and FIB-4, exhibited high diagnostic performance levels in both CHB and CHC. Furthermore, the AUROCs were similar and relatively stable among patients with serum ALT <1×, 1–2×, and ≥2× upper limit of normal (ULN) in both CHB and CHC (Supplementary Table S5). This finding suggests that the diagnostic performance levels are not apparently affected by the magnitude of hepatitis activity, as reflected by serum ALT levels. Because patients with chronic viral hepatitis, particularly CHB, tend to have fluctuating serum ALT levels over the disease course, an index that has stable performance across ALT ranges is preferred over those sensitive to ALT levels, such as APRI (Supplementary Table S5). Notably, the mFIB-4 index exhibited significantly higher performance levels than those of the FIB-4 index in both CHB and CHC. According to our statistical analysis, AST and ALT had a similar weight, with both parameters having odds ratios of approximately 1.0 in the logistic regression model. We thus decided to modify the formula of the FIB-4 index and utilise the ALT value in the denominator rather than its square root. This minor modification of the formula balances the effect of ALT relative to that of AST and considerably improves its diagnostic performance. Although FibroQ, mFIB-4, Lok index, and AARPRI exhibited high diagnostic performance levels for cirrhosis, the AUROCs of these indices were generally numerically higher in CHC patients than in CHB patients. Differences in the pathology between CHB and CHC might be responsible for the different performance levels. The underlying mechanism remains to be elucidated. Finally, despite the fact that FibroQ and AARPRI exhibited similar performance levels to those of mFIB-4, the original derivation of these two indices was not based on the logistic regression formula and therefore suffers statistical weakness.

We acknowledge several limitations in our study. First, not all patients with CHB or CHC received percutaneous liver biopsy, because of their concern about possible complications or procedure-related contraindications, such as ascites, coagulopathy, or bleeding tendency, particularly for patients with decompensated liver disease^34,35^. It may not be representative of the full spectrum of patients with chronic viral hepatitis. Second, our patients were enrolled from a single referral centre; thus, selection bias may occur. Third, even liver biopsy has the inherent issues of sampling variability and intraobserver divergence in the histological interpretation^36–38^. Finally, although the bootstrap method was undertaken for internal validation^39^, a more rigorous validation with an independent external cohort is still needed to confirm the role of the mFIB-4 index in predicting liver fibrosis in patients with chronic viral hepatitis.

In conclusion, our study demonstrated that compared with other indices, FibroQ and mFIB-4 are the two optimal diagnostic methods for predicting cirrhosis in Asian patients with CHB and CHC. Compared with mFIB-4, FibroQ and mFIB-4 exhibited similar diagnostic performance levels despite the additional inclusion of INR in the formulation of FibroQ. Thus, we propose that mFIB-4 is a simple, inexpensive, and readily available method to assess liver cirrhosis and it enables the timely implementation of surveillance programs for varices and hepatocellular carcinoma. Whether mFIB-4 can be used to monitor long-term dynamic changes in fibrosis as a result of treatment effects or disease progression remains to be studied.

## Materials and Methods

### Patients

A total of 2,681 patients with chronic viral hepatitis received liver biopsy at China Medical University Hospital (CMUH) between January 2005 and February 2016. We excluded patients (n = 250) with biopsy tissue lengths less than 1.5 cm and those (n = 155) with concurrent CHB and CHC. Finally, we enrolled 992 CHB patients and 1,284 CHC patients for further evaluation. Data on patients’ baseline characteristics and laboratory parameters, including age, sex, AST, ALT, platelet count, creatinine, and INR within 7 days before liver biopsy, were collected. This study was approved by the Research Ethics Committee of CMUH, Taichung, Taiwan (CMUH105-REC3-068). The requirement of written informed consent was waived because of the retrospective nature of the study.

### Histological assessment

Fibrosis staging was assessed according to METAVIR, which was classified as follows: F0, no fibrosis; F1, portal fibrosis without septa; F2, portal fibrosis with few septa; F3, numerous septa without cirrhosis; and F4, cirrhosis^40^.

### Noninvasive indices for liver fibrosis and cirrhosis

Various previously published noninvasive indices, including platelet count, AAR, APRI, AARPRI, FIB-4, Pohl score, AP index, FibroQ, and Lok index, were analysed to predict liver fibrosis and cirrhosis. These indices were calculated using the following formulas:

$${\rm{AAR}}\,=\,\frac{{\rm{AST}}}{{\rm{ALT}}}\,{\rm{ratio}}$$

$${\rm{APRI}}\,=\,\frac{\mathrm{AST}\,(/{\rm{ULN}})}{\mathrm{Platelet}\,\mathrm{count}\,({10}^{9}/{\rm{L}})}\,\times \,100$$

PS: The ULN for AST was 30 U/L

$${\rm{AARPRI}}\,=\,\frac{{\rm{AAR}}}{\mathrm{Platelet}\,\mathrm{count}\,({10}^{9}/{\rm{L}})/150}\,$$

$${\rm{F}}{\rm{I}}{\rm{B}}{\textstyle \text{-}}4=\frac{{\rm{A}}{\rm{g}}{\rm{e}}\,({\rm{y}}{\rm{e}}{\rm{a}}{\rm{r}}{\rm{s}})\times {\rm{A}}{\rm{S}}{\rm{T}}\,({\rm{U}}/{\rm{L}})}{{\rm{P}}{\rm{l}}{\rm{a}}{\rm{t}}{\rm{e}}{\rm{l}}{\rm{e}}{\rm{t}}\,\,{\rm{c}}{\rm{o}}{\rm{u}}{\rm{n}}{\rm{t}}\,({10}^{9}/{\rm{L}})\times \sqrt{{\rm{A}}{\rm{L}}{\rm{T}}\,({\rm{U}}/{\rm{L}})}}$$

Pohl score  = If AST/ALT less than 1 and platelets >150,000 then excludes marked fiborsis

$${\rm{AP}}\,{\rm{index}}={\rm{Age}}\,{\rm{score}}+{\rm{Platelet}}\,{\rm{score}}$$

PS: Age (years) <30 = 0; 30–39 = 1; 40–49 = 2; 50–59 = 3; 60–69 = 4; ≥70 = 5.

Platelet count (10^9^/L): ≥225 = 0; 200–224 = 1; 175–199 = 2; 150–174 = 3; 125–149 = 4; <125 = 5.

$${\rm{FibroQ}}=10\times \frac{{\rm{Age}}\,({\rm{years}})\times {\rm{AST}}\,({\rm{U}}/{\rm{L}})\times {\rm{INR}}}{{\rm{ALT}}({\rm{U}}/{\rm{L}})\times {\rm{Platelet}}\,\mathrm{count}\,({10}^{9}/{\rm{L}})}$$

$${\rm{Lok}}\,{\rm{index}}=-5.56-0.0089\times {\rm{Platelet}}\,{\rm{count}}\,({10}^{9}/{\rm{L}})+1.26\times {\rm{AST}}/{\rm{ALT}}+5.27\times {\rm{INR}}$$

### Statistical analyses

Statistical analyses were performed using SAS Version 9.4 (SAS Institute, Inc., Cary, NC, USA). Continuous variables are summarised as the median (interquartile range). Comparisons of continuous variables between two groups were conducted using the Mann–Whitney U test. Categorical variables were analysed using the chi-square test or Fisher’s exact test, as appropriate. The sensitivity, specificity, and AUROC of the noninvasive indices were obtained and compared using the ROC curve to differentiate cirrhosis (F4) or advanced fibrosis (F3) from the other fibrosis stages (for example: F0–F3 versus F4; F0–F2 versus F3–F4; F0–F1 versus F2–F4). We used the DeLong test to compare the AUROCs of two noninvasive indices. The cut-off values of the noninvasive indices were those that maximised the sum of sensitivity and specificity values (Youden Index) for the pathological diagnosis of different fibrosis stages. A *p* value less than 0.05 was considered significant. A multiple logistic regression model was used to estimate the adjusted coefficients and odds ratios for the predictors of cirrhosis. We used the independent factors of the logistic regression model and the proportions of their corresponding odds ratios to formulate the model according to the principle of parsimony^41^.

For internal validation of the model, we used bootstrapping with 1,000 replications to evaluate its AUROCs^39^. Bootstrapping is one type of resampling technique which relies on random sampling with replacement to evaluate the distribution properties of the samples and estimate the parameters derived from empirical bootstrap distribution indirectly^39^. The advantage of the bootstrap method is its convenience and efficiency to estimate the parameters of interest in developed empirical bootstrap distribution models and validate them in the original sample. This procedure has to be repeated, usually at least 200 times for the optimal stability of the results.

## Electronic supplementary material

Supplementary Information
