# Publisher Correction: New noninvasive index for predicting liver fibrosis in Asian patients with chronic viral hepatitis

**DOI:** 10.1038/s41598-018-24186-5

**Published:** 2018-04-11

**Authors:** Hung-Wei Wang, Cheng-Yuan Peng, Hsueh-Chou Lai, Wen-Pang Su, Chia-Hsin Lin, Po-Heng Chuang, Sheng-Hung Chen, Ching-Hsiang Chen, Wei-Fan Hsu, Guan-Tarn Huang

**Affiliations:** 1 Division of Hepatogastroenterology, Department of Internal Medicine, China Medical University Hospital, Taichung, Taiwan; 2 School of Medicine, China Medical University, Taichung, Taiwan; 3 School of Chinese Medicine, China Medical University, Taichung, Taiwan

Correction to: *Scientific Reports* 10.1038/s41598-017-03589-w, published online 12 June 2017

The original version of this Article contained a typographical error in the Abstract.

“The formula of the mFIB-4 index is 10 × Age(years) × AST(U/L)/Platelet count(10^9^/L) × AST(U/L).”

now reads:

“The formula of the mFIB-4 index is 10 × Age(years) × AST(U/L)/Platelet count(10^9^/L) × ALT(U/L).”

This has now been corrected in the PDF and HTML versions of the Article.

