# Supplementary material for: New noninvasive index for predicting liver fibrosis in Asian patients with chronic viral hepatitis
Source: Sci Rep. 2017 Jun 12;7:3259. doi: 10.1038/s41598-017-03589-w (PMC5468237; doi:10.1038/s41598-017-03589-w)
Supplement: Supplementary file 1 — Supplementary Information [file 41598_2017_3589_MOESM1_ESM.pdf]

Supplementary Information

New noninvasive index for predicting liver fibrosis in Asian patients with chronic viral hepatitis

Hung-Wei Wang, Cheng-Yuan Peng, Hsueh-Chou Lai, Wen-Pang Su,

Chia-Hsin Lin, Po-Heng Chuang, Sheng-Hung Chen, Ching-Hsiang Chen,

Wei-Fan Hsu & Guan-Tarn Huang

Table S1. Baseline characteristics of patients with HBV- and HCV-related cirrhosis.

ALT, alanine aminotransferase; AST, aspartate aminotransferase; CHB, chronic hepatitis B; CHC, chronic hepatitis C; INR, international normalised ratio; PT, prothrombin time.

Variables<br>Median (IQR) or n (%)	CHB<br>(n = 249)	CHC<br>(n = 208)	*p* value
Age: years	55 (18)	62 (13)	< 0.0001
Sex			< 0.0001
Female	50 (20.1)	121 (58.2)	
Male	199 (79.9)	87 (41.8)	
AST: IU/L	45 (31)	77.5 (60.5)	< 0.0001
ALT: IU/L	45 (32)	78 (79.5)	< 0.0001
Total bilirubin: mg/dL	1.08 (0.69)	1.10 (0.61)	0.9154
PT: INR	1.1 (0.2)	1.1 (0.1)	0.055
Platelet: $\times 10^3/\mu L$	117 (75)	102.5 (54.5)	0.0032
Creatinine: mg/dL	0.86 (0.28)	0.79 (0.33)	0.0156

Table S2. Univariable logistic regression model of factors associated with cirrhosis in CHB and CHC patients. ALT, alanine aminotransferase; AST, aspartate aminotransferase; CHB, chronic hepatitis B; CHC, chronic hepatitis C; INR, international normalised ratio; PT, prothrombin time.

	CHB patients (n = 992)		CHC patients (n = 1,284)	
Variables	OR (95% CI)	*p* value	OR (95% CI)	*p* value
Age: years	1.073 (1.059–1.087)	< 0.0001	1.098 (1.079–1.117)	< 0.0001
Sex (Male)	1.486 (1.047–2.108)	0.0264	0.730 (0.540–0.985)	0.0398
AST: IU/L	0.997 (0.995–0.999)	0.0039	1.004 (1.001–1.006)	0.0024
ALT: IU/L	0.992 (0.989–0.994)	< 0.0001	0.998 (0.996–1.000)	0.0408
Total bilirubin: mg/dL	1.045 (0.974–1.122)	0.2200	2.147 (1.627–2.833)	< 0.0001
PT: INR	297.029 (73.033–1208.038)	< 0.0001	15232 (2738–84735)	< 0.0001
Platelet: × 10^3/μL	0.978 (0.975–0.981)	< 0.0001	0.97 (0.966–0.975)	< 0.0001
Creatinine: mg/dL	1.025 (0.833–1.26)	0.8173	1.195 (1.003–1.424)	0.0463

Table S3. Diagnostic accuracies of noninvasive indices for predicting advanced fibrosis (F0–F2 versus F3–F4). AAR, AST/ALT ratio; AARPRI, AAR/PLT ratio index; AP index, age-PLT index; APRI, AST/PLT ratio index; CHB, chronic hepatitis B; CHC, chronic hepatitis C; FIB-4, fibrosis index based on the four factors; FibroQ, fibrosis quotient; mFIB-4, modified FIB-4; NPV, negative predictive value; PPV, positive predictive value.

Index	AUROC	Cut-off	Sensitivity	Specificity	PPV	NPV	Sensitivity+ Specificity-1
CHB							
Platelet	0.7937 (0.8092–0.8239)	155	68.9%	81.0%	69.5%	80.6%	49.9%
AAR	0.7521 (0.7111–0.7826)	0.8	70.5%	69.7%	59.5%	79.0%	40.2%
AARPRI	0.8290 (0.8448–0.8550)	0.75	74.2%	77.8%	67.8%	82.7%	52.0%
APRI	0.6191 (0.7115–0.6542)	0.7	70.5%	50.7%	47.4%	73.2%	21.2%
FIB-4	0.7869 (0.8344–0.8162)	2	67.1%	78.5%	66.2%	79.1%	45.6%
Pohl score	0.6466 (0.6309–0.6715)	0,1	33.4%	95.9%	83.7%	69.6%	29.3%
AP index	0.8014 (0.8153–0.8294)	6	68.2%	78.5%	66.6%	79.7%	46.6%
Lok index	0.8310 (0.8466–0.8571)	0.44	77.0%	78.0%	68.8%	84.4%	55.0%
FibroQ	0.8318 (0.8578–0.8572)	2.1	82.8%	68.0%	61.9%	86.3%	50.8%
mFIB-4	0.8232 (0.8577–0.8493)	2.5	70.0%	78.3%	67.0%	80.6%	48.3%
CHC							
Platelet	0.7999 (0.8092–0.8268)	140	66.5%	81.0%	63.1%	83.2%	47.5%
AAR	0.6697 (0.7111–0.7007)	0.85	61.5%	65.4%	46.4%	77.7%	26.9%
AARPRI	0.8028 (0.8448–0.8293)	0.9	67.7%	80.5%	62.9%	83.6%	48.2%
APRI	0.7729 (0.7115–0.7997)	1.4	62.5%	79.4%	59.6%	81.3%	41.8%

FIB-4	0.8425 (0.8344–0.8651)	2.3	84.1%	69.6%	57.5%	90.0%	53.7%
Pohl score	0.6265 (0.6309–0.6496)	0,1	30.2%	95.1%	75.2%	73.6%	25.3%
AP index	0.8038 (0.8153–0.8290)	8	58.9%	87.0%	68.9%	81.3%	45.9%
Lok index	0.8397 (0.8466–0.8635)	0.36	76.5%	78.9%	63.9%	87.3%	55.4%
FibroQ	0.8301 (0.8578–0.8544)	3.3	74.1%	75.9%	60.0%	85.7%	50.0%
mFIB-4	0.8152 (0.8577–0.8405)	3.4	67.5%	80.3%	62.6%	83.5%	47.8%

Table S4. Diagnostic accuracies of noninvasive indices for predicting significant fibrosis (F0–F1 versus F2–F4). AAR, AST/ALT ratio; AARPRI, AAR/PLT ratio index; AP index, age-PLT index; APRI, AST/PLT ratio index; CHB, chronic hepatitis B; CHC, chronic hepatitis C; FIB-4, fibrosis index based on the four factors; FibroQ, fibrosis quotient; mFIB-4, modified FIB-4; NPV, negative predictive value; PPV, positive predictive value.

Index	AUROC	Cut-off	Sensitivity	Specificity	PPV	NPV	Sensitivity+ Specificity-1
CHB							
Platelet	0.6962 (0.8092–0.7292)	150	46.5%	88.5%	90.8%	40.4%	35.0%
AAR	0.6803 (0.7111–0.7166)	0.8	54.4%	75.3%	84.4%	40.2%	29.7%
AARPRI	0.7349 (0.8448–0.7679)	0.75	53.3%	84.3%	89.3%	42.4%	37.6%
APRI	0.6144 (0.7115–0.6544)	0.5	82.4%	38.3%	76.6%	47.0%	20.7%
FIB-4	0.7367 (0.8344–0.7698)	1.5	69.2%	70.4%	85.1%	48.2%	39.6%
Pohl score	0.5793 (0.6309–0.5981)	1	20.0%	95.8%	92.2%	32.9%	15.9%
AP index	0.7244 (0.8153–0.7571)	5	61.8%	75.0%	85.8%	44.5%	36.8%
Lok index	0.7443 (0.8466–0.7769)	0.4	54.6%	84.4%	89.5%	43.2%	38.9%
FibroQ	0.7466 (0.8578–0.7789)	2.0	64.1%	76.7%	87.1%	46.6%	40.8%
mFIB-4	0.7390 (0.8577–0.7717)	1.8	67.8%	72.6%	85.8%	47.9%	40.3%
CHC							
Platelet	0.7741 (0.8092–0.8004)	160	58.5%	84.1%	90.1%	45.1%	42.6%
AAR	0.5462 (0.7111–0.5821)	0.7	64.9%	44.1%	74.1%	33.7%	8.9%
AARPRI	0.7086 (0.8448–0.7385)	0.7	64.0%	67.6%	83.0%	43.2%	31.6%
APRI	0.7793 (0.7115–0.8075)	0.7	74.3%	70.8%	86.3%	52.7%	45.1%

FIB-4	0.8136 (0.8344–0.8382)	2.2	64.9%	84.3%	91.1%	49.3%	49.2%
Pohl score	0.5659 (0.6309–0.5815)	1	17.0%	96.2%	91.7%	31.9%	13.2%
AP index	0.7823 (0.8153–0.8088)	6	68.5%	75.7%	87.4%	49.3%	44.2%
Lok index	0.7691 (0.8466–0.7962)	0.4	65.4%	76.2%	87.2%	47.2%	41.7%
FibroQ	0.7496 (0.8578–0.7779)	2.5	67.1%	69.2%	84.3%	46.0%	36.3%
mFIB-4	0.7368 (0.8577–0.7658)	3	53.3%	81.9%	87.9%	41.5%	35.2%

Table S5. Diagnostic accuracies of noninvasive indices for predicting cirrhosis (F0–F3 versus F4) stratified by ALT levels.

AAR, AST/ALT ratio; AARPRI, AAR/PLT ratio index; ALT, alanine aminotransferase; AP index, age-PLT index; APRI, AST/PLT ratio index; CHB, chronic hepatitis B; CHC, chronic hepatitis C; FIB-4, fibrosis index based on the four factors; FibroQ, fibrosis quotient; mFIB-4, modified FIB-4; ULN, upper limit of normal.

	ALT <1× ULN	ALT 1–2× ULN	ALT ≥2× ULN
CHB patients	n = 262	n = 311	n = 419
		AUROC (95% CI)	
Platelet	0.8299	0.7856	0.7678
AAR	0.6795	0.7696	0.7504
AARPRI	0.8356	0.8252	0.8438
APRI	0.8439	0.7945	0.7138
FIB-4	0.8472	0.8178	0.8290
Pohl score	0.7384	0.6604	0.5525
AP index	0.8038	0.7921	0.8149
Lok index	0.8110	0.8263	0.8464
FibroQ	0.8360	0.8335	0.8616
mFIB-4	0.8349	0.8226	0.8564
CHC patients	n = 278	n = 387	n = 619
		AUROC (95% CI)	
Platelet	0.8178	0.8694	0.8381
AAR	0.7593	0.8602	0.7644
AARPRI	0.8716	0.9015	0.8585

APRI	0.8435	0.8745	0.7702
FIB-4	0.9081	0.9025	0.8401
Pohl score	0.7264	0.7235	0.5985
AP index	0.8819	0.8618	0.8100
Lok index	0.8735	0.8851	0.8613
FibroQ	0.9112	0.9134	0.8694
mFIB-4	0.9057	0.9103	0.8595
